# The global impact factors of net primary production in different land cover types from 2005 to 2011

**DOI:** 10.1186/s40064-016-2910-1

**Published:** 2016-08-02

**Authors:** Bo Yu, Fang Chen

**Affiliations:** 1 Key Laboratory of Digital Earth Science, Institute of Remote Sensing and Digital Earth, Chinese Academy of Sciences, Beijing, 100101 China; 2 Hainan Key Laboratory of Earth Observation, Institute of Remote Sensing and Digital Earth, Chinese Academy of Sciences, Sanya, 572029 China

**Keywords:** NPP, Random forest, Land cover type, Anthropogenic impact

## Abstract

With the seriously polluted environment due to social development, the sustainability of net primary production (NPP), which is used to feed most lives on the earth, has become one of the biggest concerns that we have to consider for the sake of food shortage. There have been many researches analyzing one or two potential impact factors of NPP based on field observation data, which brings about many uncertainties for further calculation. Moreover, the frequently used process-based models heavily depend on the understandings of researchers about the NPP process. The premises of such models hinder the impact factor analysis from being objective and confident. To overcome such shortages, we collected 27 potential impact factors of global NPP in terms of eight land cover types. The feature variables include atmosphere, biosphere, anthroposphere and lithosphere parameters, which can be obtained from public available remote sensed products. The experiment shows that latitude, irradiance ultraviolet and normalized difference vegetation index are dominant factors impacting global NPP. Anthropogenic activities, precipitation and surface emissivity are influencing NPP calculation largely. However, some commonly used biosphere parameters in process-based models are actually not playing that important roles in NPP estimation. This work provides a new insight in analyzing NPP impact factors, being more objective and comprehensive compared with frequently used process-based models.

## Background

The increasing global population, energy consumption and the frequent natural disasters are drawing more and more attention about the future sustainability of natural resources. There are concerns that the natural resources are beyond the safety margin of feeding human beings and other animals with the influence of anthropogenic activities (Rockström et al. 2009). Apart from human impact, the natural biogeochemical cycles also lead to the change of troposphere concentrations of greenhouse gases (Xiao et al. 1995) and solar radiation. The resultant climate change, together with the changes of temperatures, concentrations of greenhouse gases, may drive a significant variation on the biogeochemical process of the terrestrial ecosystems (Melillo et al. 1990; Gates 1985; Houghton and Woodwell 1989; Jenkinson et al. 1991). The global biomass productivity is a critical limiting resource (Running 2012), which is limited by land area, water, soil moisture and solar radiation.

Net primary production (NPP) measures the net amount of carbon assimilated after photosynthesis and autotrophic respiration over a given period of time (Clark et al. 2001). It harbors most species in the earth by providing food sources and services. Moreover, NPP is related to policy making. As described in the economic-emission model (Dean et al. 1992), the anthropogenic emissions, anthropogenic impacts on ecosystems and their corresponding NPP aggregations are linked with each other (Xiao et al. 1995). Therefore, monitoring NPP is of significantly important for maintaining sustainable natural resources for most species on earth. Better understanding of NPP impact factors helps develop management plans for land planning to satisfy human requirements and minimize the impact of climate change (Smith et al. 2012).

There are multiple researches analyzing the impact factors of NPP, such as precipitation, temperature and atmospheric carbon dioxide concentration (Melton et al. 2013). Some researchers conclude that soil moisture is the dominant factor controlling NPP compared with annual rainfall (Raich et al. 1991). NPP of arid ecosystem is mostly influenced by water, by enhancing the water use efficiency of vegetation (Xiao et al. 1995). For areas with limited temperature, NPP will get larger when temperature becomes higher (Nemani et al. 2003; Lucht et al. 2002; Bogaert et al. 2002; Piao et al. 2006). In terms of tropical areas, solar radiation becomes the dominant factor in influencing NPP because of severe cloudiness (Zhao and Running 2010; Garbulsky et al. 2010). The annual NPP is strongly correlated with the mean annual precipitation and temperature in dry and cold areas (Zhu and Southworth 2013; Schuur 2003). Moreover, there are also several studies suggesting a significant correlation relationship between normalized difference vegetation index (NDVI) and NPP (Paruelo et al. 1997; Fensholt et al. 2013). NDVI, as a difference ratio of near infra-red and red reflectance values, is commonly used to monitor vegetation growth status and the vegetation coverage.

For global NPP, different climatic characteristics of different types of land are influenced by different variables. Generally, the land types cover forest, water, shrub lands, cropland, permanent wetland, grassland, built-up areas and snow cover. In terms of NPP in different types of land, there have been many researches analyzing the impact factors of NPP in each land type. Climate variables such as temperature, precipitation, topography and soil type have been validated to have strong correlation relationship with the NPP in forest (Whittaker 1970; Woodward et al. 2004; Holdridge 1964; Pan et al. 2013), savanna (Zhu and Southworth 2013; Schuur 2003), grassland (Scholes and Hall 1996; Xia et al. 2014) and wetland (Birkett 1998). The impact of anthropogenic activities in CO_2_ emissions and land cover change on NPP analyzed in studies (Prentice et al. 2001; Ou et al. 2015; Haberl et al. 2007) has turned out to be pervasive and serious.

However, the analyses above are mostly based on field data, which need to parameterize, calibrate and validate NPP estimation models (Zheng et al. 2003). The field observing data have three main disadvantages: (1) the experiment sites are selected subjectively and the field data are quite unbalanced, data collected in developed areas are more intense and accurate than that in developing areas or less-developed areas; (2) up-scaling the field data to global estimation models brings about more uncertainties, especially under the premises of different conditions for various models (Law et al. 2006; Xiao et al. 2011); (3) data sharing is a critical issue that hinders many researchers from analyzing more complex circumstances. To avoid such shortages, more and more researches start to apply remote sensed products in analyzing NPP impact factors (Potter et al. 2003; Nayak et al. 2010; Zhao et al. 2005). Moreover, the remote sensed NPP product has been proved to be able to support real estimation of the process of carbon cycle and available to validate NPP monitoring after a recent comparison between the estimates from a dynamic vegetation model and remote sensed NPP products.

Models in calculating NPP are generally grouped into process-based model and data-driven regression model. The process-based model is designed based on different understandings of the process, at a local scale by synthesizing soil, temperature, climatic variables to calculate NPP. Much calibration needs to be done with observations to up-scale such model to study a large region (Li et al. 2014). However, current studies indicate poor performances of process-based model with large differences between observation values and model estimations (Schwalm et al. 2010). Data-driven regression model is limited by the amounts of data provided to train the regression model, which can be overcome by large quantities of remote sensed products. Random forest (Breiman 2001) has been used (Tramontana et al. 2015) in predicting annual gross primary production (GPP) and found that remote sensed data drives the model close to the optimum.

Since publicly acceptable impact factors of global NPP have not been analyzed widely, we investigate the potential impact factors of global NPP spatial pattern in terms of different land cover types. Firstly, a random forest regression model is trained to estimate NPP spatial pattern for each land cover type based on all the concerning climatic variables, soil temperature, moisture, anthropogenic emissions and consumptions. Process-based model is not used here, because it is limited by its understanding of NPP process. Secondly, on top of each trained model, we get importance ranking of all the variables for each type of land. Thirdly, the importance rankings are further used to investigate main impact factors for each type of land globally and provide supporting data for policy makings. To the best of our knowledge, this work first quantifies the impact of each potential factor in estimating NPP to provide an objective analysis for each type of land globally. The group of potential impact factors first covers a wide range of variables, comprising atmospheric parameters, climatic parameters, soil and anthropogenic consumptions.

## Methods

The experimental data in this paper cover climate data, biosphere data and anthropogenic energy consumption. In terms of global NPP pattern calculation, we have generated and downloaded the concerning annual global remote sensed products mainly from http://giovanni.sci.gsfc.nasa.gov/giovanni/ and http://gdata1.sci.gsfc.nasa.gov/daac-bin/G3/gui.cgi?instance_id=neespi, managed by NASA GES DISC (Acker and Leptoukh 2007). All the remote sensed products in this study are resampled to 1° × 1°.

### Climate and biosphere data

As shown in Table 1, the feature variables indexed from 3 to 22 are climate and biosphere data. Planetary boundary layer height above surface (Wikipedia 2015), soil temperature (NASA 2013), soil moisture (NASA 2015), land surface temperature, aerosol index (OMI 2006), wind speed, precipitation (NASA 2015), irradiance ultraviolet (Montzka et al. 2011), net all-wave radiation (NASA 2014) and spectral indexes are all frequently used parameters in NPP calculation (Melillo et al. 1990; Dean et al. 1992; Woodward et al. 2004; Birkett 1998). The concentrations of tropospheric NO_2_ and CO_2_ are also used in our study as atmosphere parameters, impacting NPP as well (Xiao et al. 1995). There has been research indicating that biomass burning influences soil fuel and atmospheric environment (Freeborn et al. 2011; Boschetti and Roy 2009). Therefore, fire radiative power (FRP), i.e. the emissions of fire radiative energy, as an indicator to quantify biomass burning and trace gases, is used to express fire intensity in this work. Furthermore, surface emissivity, plant canopy surface water and canopy water evaporation are used as potential impact factors in NPP calculation.

**Table 1:** Feature variables and their corresponding indexes used for calculating NPP

Feature index	Feature variable
1	Longitude
2	Latitude
3	Aerosol index
4	Carbon dioxide
5	GPCP (global precipitation climatology project) precipitation rain rate
6	Planetary boundary layer height above surface (height)
7	Nitrogen dioxide
8	Soil temperature
9	Soil moisture
10	Enhanced vegetation index
11	Fire radiative power
12	Land surface temperature in the night time
13	Land surface temperature in the daytime
14	Normalized difference vegetation index (NDVI)
15	Irradiance ultraviolet
16	Net longwave radiation
17	Net shortwave Radiation
18	Aerosol optical depth
19	Surface emissivity
20	Plant canopy surface water
21	Canopy water evaporation
22	Wind speed
23	Oil consumption
24	Gas consumption
25	Renewable energy consumption
26	Coal consumption
27	CO_2_ consumption

As shown in research (Ardö 2015), moderate resolution imaging spectro-radiometer (MODIS) NPP product from ftp://ftp.ntsg.umt.edu/pub/MODIS/NTSG_Products/MOD17/GeoTIFF/MOD17A3/GeoTIFF_30arcsec/ is used as ground truth for training our regression model and evaluating its performance.

### Anthropogenic consumption

BP statistical Review of World Energy (2015) is the statistical review on world energy markets, published by BP, one of the world’s leading international oil and gas companies. It records annual primary energy consumption comprising oil, coal and natural gas. The consumption of renewable energy, such as solar and wind energy is recorded as well. Moreover, CO_2_ emission is published in the statistical review to reflect the anthropogenic impact on atmosphere. Each kind of statistical data is mapped to a global map with the help of ArcGIS software at the spatial resolution of 1° × 1°. In total, we have five anthropogenic products, indexed as 23–27 in Table 1.

### Random forest regression modeling

Random forest (Breiman 2001) is an ensemble learning model, composed of multiple trees. Each tree samples a different bootstrap sample of data for construction. A fixed number of features are randomly selected to train the criteria of each node. Random Forest has become a widely used machine learning model, since it performs better than support vector machines and neural networks in many researches (Liaw and Wiener 2002). The whole process of training a random forest regression model is listed as below:

1. Given training sample set S, test set T and the feature vector F of each sample. The number of trees in the forest is N and f is the fixed number of feature vector, selected from F used to train each note;
2. For each of the bootstrap samples, train a regression tree where each node is split by choosing the best criteria among the randomly selected f features at that node;
3. Obtain the regression value as the average of the N regression values from N trees.

### Variable importance calculation

Variable importance, measuring the interaction of each variable with all the others, can be obtained from a trained random forest regression model. The importance of each variable is calculated by evaluating the average error increase when it is permuted while all the other variables are maintained the same. From the calculated variable importance, we can investigate the impact of each feature variable has in the regression model. The details in calculating variable importance are described in the following equations.

1 $$VI(F_{i} ) = \frac{{\sum\nolimits_{t = 1}^{{N_{tree} }} {VI^{t} (F_{i} )} }}{{N_{tree} }}$$

2 $$VI^{t} (F_{i} ) = R_{error} (\hat{F}_{i} ) - R_{error} (F)$$

where VI(F_i_) is the variable importance of feature F_i_, VI^t^(F_i_) is the variable importance of tree t, $R_{error} (\hat{F})$ is the regression error after permuting feature F_i_ and R_error_(F) is the regression error before permuting.

### Land cover classification

This paper is focused on analyzing potential impact factors of NPP in terms of different land cover types. MODIS global land cover product in the IGBP land cover type classification is downloaded from http://glcf.umd.edu/data/lc/. The product has 18 types of land totally, but some are merged in our study for the sake of analysis. Generally, we have eight classes in our study, forest, shrub lands, savannas, grasslands, permanent wetlands, croplands and built-up area, snow and ice and barren or sparsely vegetated area. The reason why croplands and built-up area are merged together is that urban areas are typically built on or near the most productive agricultural lands. They have strong interaction between each other. The class ‘water’ is not considered in our study, because our research mainly focuses on global terrestrial ecosystem. Therefore, eight classes of land cover with eight class labels are generated, and the corresponding annual product for each land cover can be achieved by masking using each class label.

## Results

### Regression performance of random forest model

**Fig. 1:**
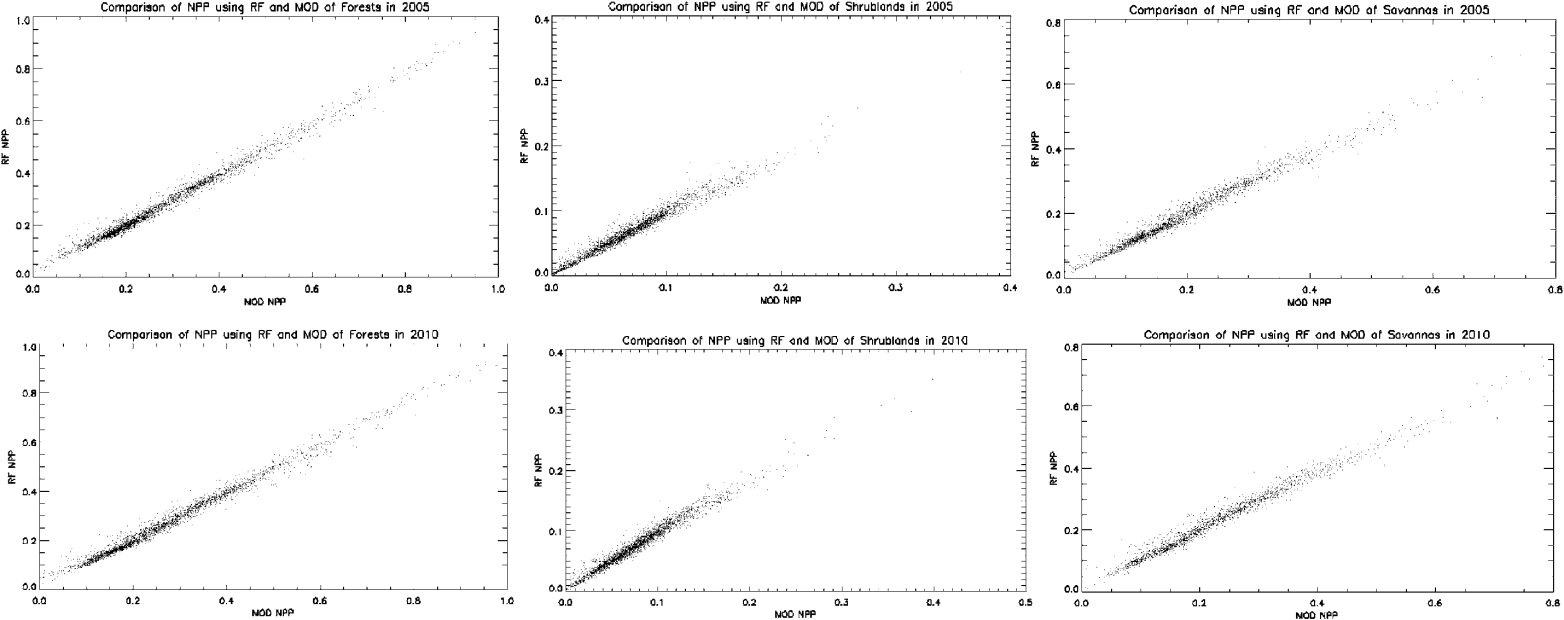
*Scatter plots* of NPP from RF and MODIS in forests, shrub lands and savannas in randomly selected test year 2005 and 2010

Of all the 7-year products of each type of land from 2005 to 2011 in this study, we randomly picked 2-year products as test data; the other 5-year products are used for training a random forest regression model to calculate global terrestrial NPP for each type of land. The ratio between the number of training and testing data is 5:2 in our case, which satisfies the commonly used pipeline in solving machine learning problems that the ratio between the quantity of training and testing data is larger than 2:1. Table 2 demonstrates the correlation coefficient of data in each test year between the estimated NPP spatial pattern and ground truth MODIS NPP in different land cover type. Furthermore, to demonstrate the correlation visually, the scatter plots of estimated NPP by random forest (RF) and MODIS NPP in three main land cover types, forests, shrub lands and savannas are shown in Fig. 1.

**Table 2:** Correlation coefficient between calculated NPP by random forest and MODIS NPP in global different types of land

Class year	Forests	Shrub lands	Savannas	Grasslands	Permanent wetlands	Croplands and built-up area	Snow and ice	Barren and sparsely vegetated
2005	0.991651	0.983342	0.988376	0.991347	0.984758	0.987507	0.991462	0.987169
2010	0.990842	0.982202	0.987927	0.991595	0.983005	0.988258	0.992126	0.984349

Concerning Table 2 and Fig. 1, the NPP pattern estimated by random forest correlates quite well with that from MODIS in each type of land. All the correlation coefficients are higher than 0.98, indicating strong correlation. The results validate that random forest model estimates NPP pattern as well as MODIS product.

### Feature variable importance of each type of land

**Fig. 2:**
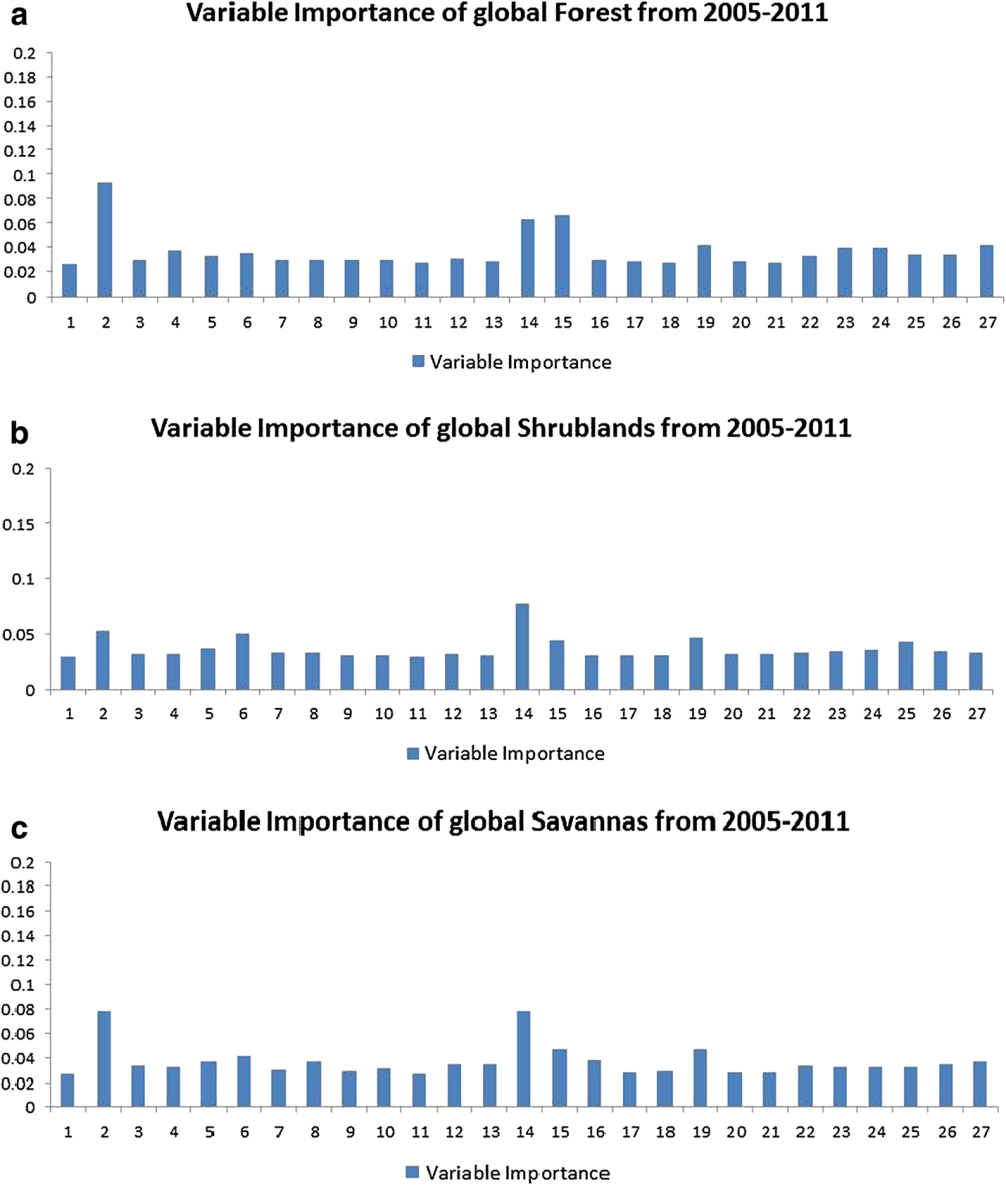
Variable importance of each factor impacting NPP in each type of land from 2005 to 2011

**Fig. 3:**
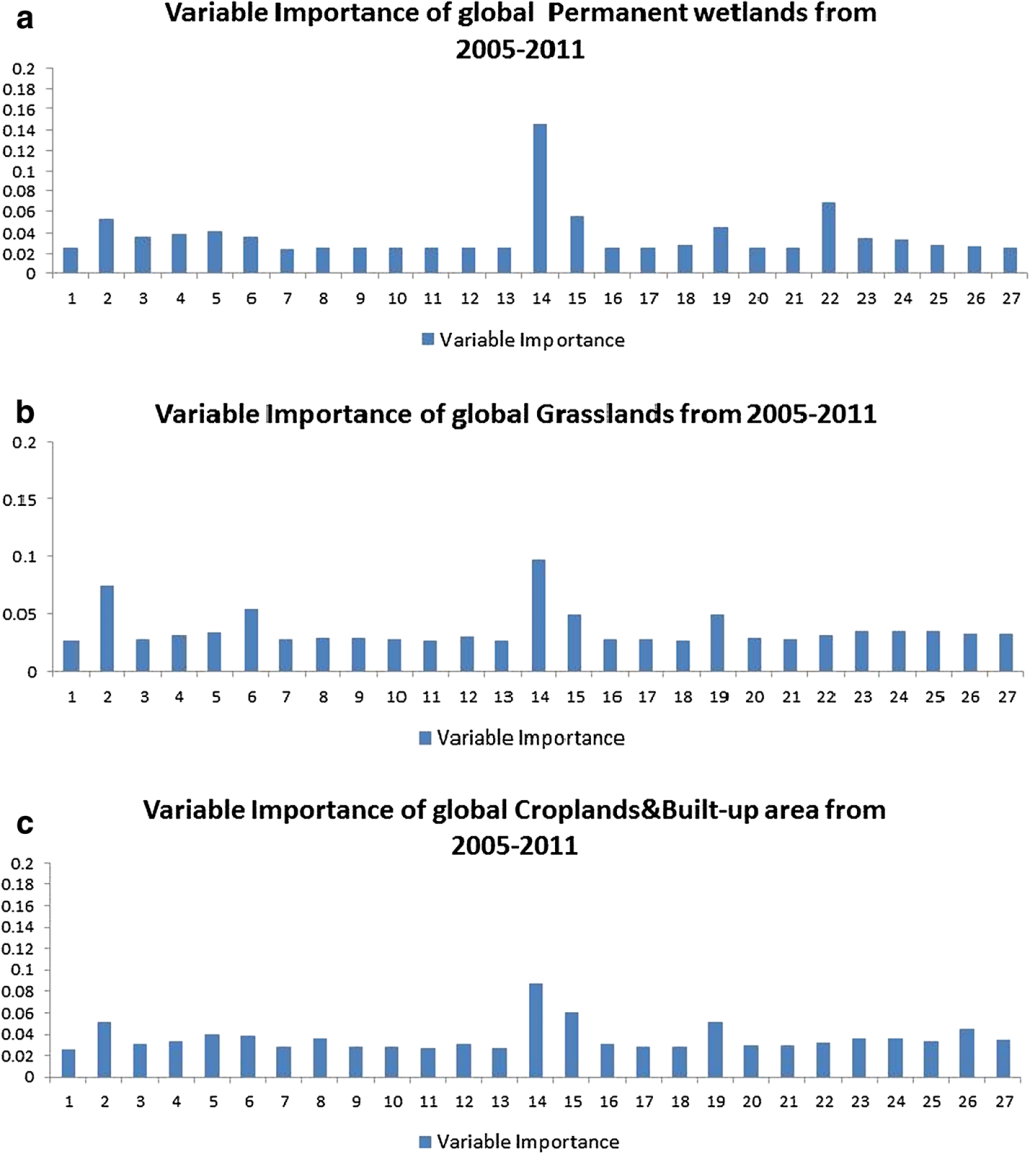
Variable importance of each factor impacting NPP in each type of land from 2005 to 2011

**Fig. 4:**
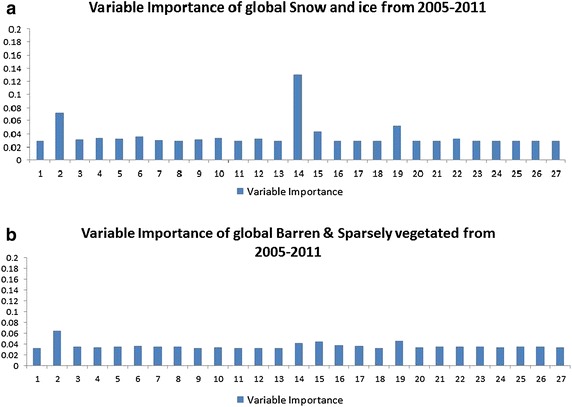
Variable importance of each factor impacting NPP in each type of land from 2005 to 2011

To further understand the importance of each feature variable in estimating NPP pattern in each type of land, we calculated variable importance for each feature on top of the trained model and demonstrated in terms of each type of land in Figs. 2, 3 and 4. The numbers are in accordance with the indexes in Table 1.

Generally, Figs. 2, 3 and 4 show that variable importance rankings in different types of land are different. For the sake of understanding the importance ranking of feature variables in the eight types of land, as shown in Figs. 2, 3 and 4, we have divided the importance rankings into four groups and demonstrated in Table 3.

**Table 3:** Importance ranking group of each land cover type in Figs. 2, 3 and 4

Forests (Fig. 2a)	1st	Latitude and irradiance ultraviolet
	2nd	NDVI
	3rd	Height, CO_2_ and anthropogenic impact
	4th	Others, such as aerosol index, NO_2_, soil temperature, soil moisture
Shrub lands (Fig. 2b)	1st	NDVI
	2nd	Latitude, height, irradiance ultraviolet, surface emissivity
	3rd	Anthropogenic impact, precipitation
	4th	Others, such as aerosol index, NO_2_, soil temperature, soil moisture
Savannas (Fig. 2c)	1st	Latitude and NDVI
	2nd	Irradiance ultraviolet and surface emissions
	3rd	Precipitation, height, soil temperature, land surface temperature, wind speed and anthropogenic impact
	4th	Others
Grasslands (Fig. 3a)	1st	Latitude and NDVI
	2nd	Height, irradiance ultraviolet and surface emissivity
	3rd	CO_2_, precipitation, wind speed and anthropogenic impact
	4th	Others, such as soil temperature, FRP, land surface temperature
Permanent wetlands (Fig. 3b)	1st	NDVI
	2nd	Wind speed
	3rd	Latitude, irradiance ultraviolet, surface emissivity and precipitation
	4th	Others, anthropogenic impact belongs to this group
Croplands and Built-up area (Fig. 3c)	1st	NDVI
	2nd	Irradiance ultraviolet, latitude and surface emissivity
	3rd	Precipitation, height, soil temperature, anthropogenic impact
	4th	Others, including soil moisture, FRP, all-wave radiation
Snow and ice (Fig. 4a)	1st	NDVI
	2nd	Latitude
	3rd	Irradiance ultraviolet and surface emissivity
	4th	Others, including anthropogenic impact
Barren & Sparsely vegetated (Fig. 4b)	1st	Latitude
	2nd	NDVI, irradiance ultraviolet, surface emissivity
	3rd	Others
	4th	–

As shown in Table 3, each type of land has four groups of feature variables, ranked one after another. For example, NPP in global forests (Fig. 2a) is mostly influenced by the two feature variables in the 1st group, latitude and irradiance ultraviolet. They determine solar radiation, which play a significant role in photosynthesis. NDVI belongs to the 2nd group, indicating the growing motion of forests. The 3rd group consists of height, CO_2_ and anthropogenic impact. Height is important in NPP estimation for forests, since it describes the growth stage of the tree. Anthropogenic impact comprises anthropogenic primary energy consumption, renewable energy consumption and CO_2_ emissions. With elevated CO_2_ concentrations, water use efficiency of the plant is enhanced, especially in high latitude ecosystems (Xiao et al. 1995). Therefore, the rate of decomposition of organic matter is increased, so that NPP is influenced. The other parameters, such as aerosol index, NO_2_, soil temperature, soil moisture and so on are actually not playing that important role in calculating NPP as expected. Moreover, most researches (Clark et al. 2001; Schuur 2003; Pan et al. 2013) conducted in analyzing forest NPP only focus on precipitation, temperature and a few other commonly used soil conditions. As far as we are concerned, there are few researches analyzing impact factors of forest NPP considering not only anthropogenic impact and solar radiation impact, but also biosphere impact, like this paper. The researches mostly focus on precipitation and temperature (Melton et al. 2013; Scholes and Hall 1996). The situation goes the same with the other seven land cover types.

## Discussion and conclusions

In this paper, we have analysed potential impact factors of global NPP in eight types of land, based on a trained random forest regression model. The regression model calculates NPP fairly well as MODIS NPP product. From the feature variable importance analysis, we find that the importance rankings of the potential impact features for different types of land are different. A systematic comparison of feature importance is done for eight types of land at a global scale and generally can be grouped into anthropogenic impact, solar radiation impact and biosphere impact.

### Solar radiation impact

Solar radiation factors, latitude and irradiance ultraviolet are ranked in the first two groups of the eight types of land. That indicates impact of solar radiation on NPP calculation is stronger than anthropogenic impact and most biosphere impact, except for NDVI.

### Anthropogenic impact

Anthropogenic impact comprises anthropogenic oil consumption, gas consumption, coal consumption, renewable energy consumption and CO_2_ emissions. They are significantly influencing NPP in most land cover types, except for permanent wetlands, snow and ice, and barren and sparsely vegetated area, which accords with the report in (Haberl et al. 2007). The anthropogenic activities in permanent wetlands, snow and ice and barren and sparsely vegetated areas are comparatively less frequent than in other types of land.

### Biosphere impact

NDVI is leading the impact that all the concerning features have on calculating NPP, together with latitude and irradiance ultraviolet. It further accords with the conclusions that NDVI is significantly correlated with NPP (Schloss et al. 1999), and NDVI has been used to replace NPP to analyze ecological cycle in many researches (Schloss et al. 1999). Apart from NDVI, surface emissivity and precipitation are influencing NPP largely in most land cover types, except for forest, which is covered by intense high trees. That comes to the same conclusion with the researches analyzing the impact factors of savannas, shrub lands and so on (Melton et al. 2013; Zhu and Southworth 2013; Schuur 2003; Scholes and Hall 1996). Moreover, FRP in this study shows little impact on global NPP. But there have been many researches concluding that FRP generates precursor of tropospheric ozone (Monks et al. 2015), which has been proved to be highly correlated with NPP to a large extent (Ainsworth et al. 2012). That indicates that the impact of FRP on NPP is not immediate, which cannot be measured using the simultaneous products. It will take a long time for the influence to become obvious.

The other biosphere impact factors, such as soil moisture, aerosol index and land surface temperature, are expected to play a dominant role in calculating NPP, while most features play limited role in effecting NPP as they rank the last group in all the land cover types. That is different from the common principle that the biosphere impact factors are important in measuring NPP and form the basic equations of NPP calculation (Vorosmarty and Schloss 1993) in process-based models. Data-driven model focuses on the importance of each feature has in calculating NPP, and constructs a synthesized model to calculate NPP. Each feature variable has a different weight, representing its importance. However, process-based model focuses on the physical understanding of the NPP generation process, which may miss more significant feature variables and may be subjective misunderstandable. The main different roles of biosphere factors playing in process-based models and data-driven models is that biosphere factors form the basic formulations in generating NPP in common belief and experience, while data-driven model calculates the significance of each feature objectively without much prior knowledge.

Since there are few researches in understanding the 27 potential impact factors of NPP pattern in different land cover types simultaneously, this study conducts a comprehensive analysis of potential impact factors in estimating global NPP spatial pattern in terms of eight types of land. We come to the conclusion that NDVI, irradiance ultraviolet and latitude are the most significant features for each type of land, which accord with many published correlation analyses. However, the commonly used features in process-based model for calculating NPP, such as soil moisture, soil temperature and aerosol index are comparatively less important than anthropogenic impact and solar radiation impact. That indicates that more relevant parameters should be considered in process-based models and they should be given objective weights in different types of land. As a complementary of process-based model, this work broadens the method in understanding ecological process system by providing an objective and effective data-driven model using publicly available remote sensed products. Moreover, the specific analysis of the impact factors on NPP may provide a direction for policy makings to decrease the anthropogenic impact and enhance NPP.

## Data accessibility

Remote sensed products: http://giovanni.sci.gsfc.nasa.gov/giovanni/, http://gdata1.sci.gsfc.nasa.gov/daac-bin/G3/gui.cgi?instance_id=neespi, http://glcf.umd.edu/data/lc/.

BP statistical review data: http://tools.bp.com/energy-charting-tool.aspx.

ArcGIS: http://www.esrichina-bj.cn/softwareproduct/ArcGIS/ArcGis%2010.1/.
